# Longitudinal Model Building Using Latent Transition Analysis: An Example Using School Bullying Data

**DOI:** 10.3389/fpsyg.2018.00675

**Published:** 2018-05-08

**Authors:** Ji Hoon Ryoo, Cixin Wang, Susan M. Swearer, Michael Hull, Dingjing Shi

**Affiliations:** ^1^Department of Educational Leadership, Foundations, and Policy, University of Virginia, Charlottesville, VA, United States; ^2^Department of Counseling, Higher Education, and Special Education, University of Maryland, College Park, College Park, MD, United States; ^3^Department of Educational Psychology, University of Nebraska-Lincoln, Lincoln, NE, United States; ^4^Department of Psychology, University of Virginia, Charlottesville, VA, United States

**Keywords:** latent transition analysis, student-centered concerns about bullying, LTA with covariates, middle and high schools, model building

## Abstract

Applications of latent transition analysis (LTA) have emerged since the early 1990s, with numerous scientific findings being published in many areas, including social and behavioral sciences, education, and public health. Although LTA is effective as a statistical analytic tool for a person-centered model using longitudinal data, model building in LTA has often been subjective and confusing for applied researchers. To fill this gap in the literature, we review the components of LTA, recommend a framework of fitting LTA, and summarize what acceptable model evaluation tools should be used in practice. The proposed framework of fitting LTA consists of six steps depicted in Figure 1 from step 0 (exploring data) to step 5 (fitting distal variables). We also illustrate the framework of fitting LTA with data on concerns about school bullying from a sample of 1,180 students ranging from 5th to 9th grade (mean age = 12.2 years, SD = 1.29 years at Time 1) over three semesters. We identified four groups of students with distinct patterns of bullying concerns, and found that their concerns about bullying decreased and narrowed to specific concerns about rumors, gossip, and social exclusion over time. The data and command (syntax) files needed for reproducing the results using SAS PROC LCA and PROC LTA (Version 1.3.2) (2015) and Mplus 7.4 (Muthén and Muthén, 1998–2015) are provided as online supplementary materials.

## Introduction

Since the early 1990s, latent transition analysis (LTA; Collins and Wugalter, 1992) has received more attention among researchers as an effective statistical analytic tool for a person-centered approach using longitudinal data (Bye and Schechter, 1986; Collins and Wugalter, 1992; Bergman and Magnusson, 1997; Masyn, 2013). However, model building in LTA has often been subjective, which is confusing for applied researchers regarding which procedures to follow, which results to report, and how to interpret the validity of their solution. It is important to examine why and where such subjectivity occurs and to discuss the best way to avoid such confusions, which is the focus throughout this paper. We first begin with how LTA can be characterized from other latent variable models and then further discuss what model specification within LTA can be related to such subjectivity and confusion and how to avoid them. All of these activities were applied with an empirical study within the context of bullying research.

Due to the subjectivity of the decision making rule in utilizing parameters of LTA and the common practice of restructuring response options when dealing with categorical response variable, model building in LTA often depends on the researcher's intentions or goals. Although there are several model building procedures described in this study, a unified framework of building a LTA model is less clear in the literature. Because model building is linked to the internal validity of a study, it is important and necessary to provide guidelines by synthesizing model building procedures.

According to the Centers for Disease Control and Prevention in the United States, bullying is defined as “any unwanted aggressive behavior(s) by another youth or group of youths” which involves a “perceived power imbalance and is repeated multiple times or is highly likely to be repeated.” Bullying has been found to lead to harmful physical, psychological, social, or educational consequences for the targeted individual (Gladden et al., 2014, p. 7). Many students are concerned about bullying and school safety, which has been found to contribute to school refusal and avoidance of school related activities (Randa and Wilcox, 2010). However, few studies have examined students' concerns about bullying using longitudinal data.

In order to fill these two gaps in the literature (lack of research on model building in LTA and on bullying concerns using longitudinal data), we review technical components of LTA modeling for researchers who are new to LTA, recommend a synthesized framework for fitting a LTA from the literature, and illustrate how to use this new framework with data on adolescents' concerns about school bullying. Using LTA with longitudinal data to study bullying concerns can help educators and school mental health professionals better understand the developmental changes in students' concerns about bullying and design more effective bullying prevention programs that address students' concerns about bullying.

## When to use LTA

In the areas of social and behavioral sciences, factor analysis has been a long-standing analytic strategy to understand unobserved (or latent) constructs as well as their internal structure from observed data (Cudeck and MacCallum, 2007). When the unobserved constructs are introduced using factor analysis, researchers are not only able to define the construct that is measured by observed variables but also to account for the measurement errors in the observed variables. In the process of modeling the unobserved constructs, it is necessary to select between latent trait models and factor analytic models according to the types of observed variables; categorical vs. continuous, respectively (Lubke and Muthén, 2005; Lubke and Miller, 2015), although both have been shown to be statistically equivalent models in certain conditions (e.g., see Takane and de Leeuw, 1987; Reise, 2012). Latent trait models are often differentiated from factor analytic models and are also called an item response model because latent trait models deal with categorical observed variables (e.g., binary response such as “Yes” or “No”; ordered polytomous response such as “Never,” “Once or twice per month,” “About once a week,” or “Several times a week”). However, both latent trait and factor analytic models have the common characteristic that the underlying latent variable (the scale) is assumed to be continuous. In this manuscript, we aim to further differentiate other types of latent variable modeling techniques, specifically, latent class analysis (LCA; Lazarsfeld and Henry, 1968) and its longitudinal version, latent transition analysis (LTA; Collins and Wugalter, 1992), from factor analysis and latent trait models. Both LCA and LTA have been applied to problems in the social and behavioral sciences, education, and public health. Some exemplary papers can be found in social and behavioral sciences (Bergman and Magnusson, 1997; Todd and Houston, 2013; Jagenow et al., 2015; Sagoe et al., 2017), medicine and public health (Guo et al., 2009; Cochran et al., 2015; Cosden et al., 2015; Kenzik et al., 2015), and education (Goldweber et al., 2011; Schweizer et al., 2014; Williford et al., 2014; Ryoo et al., 2015; Liu et al., 2016). LCA and LTA have been often called, “person-centered analyses” because LCA and LTA use response patterns of observed variables to assign individuals to unobserved latent groups (Bye and Schechter, 1986; Collins and Wugalter, 1992; Bergman and Magnusson, 1997; Masyn, 2013). A complement to person-centered analyses in latent variable modeling is the latent trait and/or factor analytic models, which are variable-centered analyses.

To illustrate when it is appropriate to use LCA and LTA instead of factor analysis, we use the example of student-centered concerns about bullying collected from a self-report questionnaire with six items (e.g., “How concerned or afraid are you that you might be physically attacked or hurt by another student or a group of students”) on the Pacific-Rim Bullying measure (PRBm; Konishi et al., 2009) which uses a 4-point Likert-type format for each item ranging from 1 (*No, not at all*) to 4 (*Yes, very much*). Utilizing a person-centered approach, LCA, we address specific research questions such as: (a) Are there qualitatively distinct subgroups of students who demonstrate particular patterns of concerns regarding bullying? (b) What variables predict each student's group membership/status based on their concerns? For example, some students are seriously concerned about physical bullying, but not at all concerned about relational bullying and vice versa.

It is also natural to extend such person-centered analysis into a longitudinal context using LTA, a longitudinal version of LCA. In longitudinal data analysis, the primary goal is to examine the change over time and to identify the association of repeated measures using a variance-covariance matrix (Long, 2011). In LTA, participants' corresponding memberships could change over time, because people change. Thus, an additional interesting empirical question might arise in LTA: (c) Do participants stay in the latent group to which they were assigned, or do they transition to a different latent group at a later time point? Based on student's membership change over time, it is also possible to trace the trend of change on the concerns at the student level. That is, LTA allows researchers to characterize the memberships [Questions (a) and (b)] as well as to predict changes among memberships [Question (c)]. In addition to research questions directly linked to model parameters, we can further investigate the relationship among group characteristics based on students' concerns and other variables such as: (d) Is the change in latent group status affected by a student's grade? Since each student is assigned into one latent group in both LCA and LTA, we can interpret each student's concerns about bullying and further examine the associations of his or her status of bullying concerns with other variables of interest that were not used in forming latent classes.

## Literature review on model selection in LTA

Model building is an important research topic in LTA because there is still a lack of unified method in the methodological literature. The lack of unified method on model building is directly related to the complexity of the model and its evaluation. We list two specific reasons that do not only correspond to unique properties of LTA specification, but are also related to a subjective decision making rule. The first is that enumeration of latent statuses in LTA is often involved with content-specific decision making. That is, enumerating latent statuses is not only based on identifying homogeneous groups using fit indices or the result of a hypothesis test, but it is also determined by the content-specific and theoretical foundations often represented by response patterns. For example, a latent status made-up of only 1–2% of participants may not be representative; or two latent statuses that are only different in a low discriminant item (its probabilities for classes were similar) need not to be classified even if those are suggested by fit indices or a statistical test/procedure.

The second important reason would be restructuring response categories to avoid model non-identification issues which is then related to estimation problems. When specifying an LTA model, the number of parameters is countable and identifiable with a model formula of LTA. Thus, model identification may be regarded as a simple step by calculating degrees of freedom defined by *df* = *W* − *P* − 1, where *W* is size of cells and *P* is the number of parameters. In other words, as long as a model is identified (i.e., positive degree of freedom), it is expected to estimate parameters using the maximum likelihood (ML) method. However, there is no guarantee in LTA that a model identified will not have any estimation issues because *W* is not equal to sample size due to large number of incomplete cells. Collins and Lanza (2010) indicate that if the ratio of given data (*N* = sum of frequencies in non-empty cells) to unknown parameters decreases, LCA and LTA can be under-identified or even unidentified with positive degrees of freedom. Thus, to increase the precision of ML estimates, researchers are asked to reduce the response categories because reducing response categories results in smaller number of parameters. For example, in the example data about concerns about bullying, a contingency table from (4^6^)^3^ = 68, 719, 476, 736 cells that comprise the raw data coming from four options of six items over three time points would be used to estimate model parameters. The goal of fitting a LTA is to represent the contingency table into interpretable classes. Due to the large number of cells that comprise the data and the likelihood of having empty cells, model identification issues abound. So, to reduce this issue in the current context and as done in several studies that have applied LTA, researchers collapsed their data into smaller number of responses. In this study, since we are mainly interested in patterns of what adolescents were concerned about bullying and less interested in the degree to which they were concerned, we dichotomize the four categories into two categories: “*No, not at all*” and “*No, not much*,” were merged into the no concern category (coded as 0) and the other two categories, “*Yes, a little*” and “*Yes, very much*,” were merged into the concern category (coded as 1). As a result of this dichotomizing, the cell size significantly decreased to (2^6^)^3^ = 262, 144, which helped us to avoid model identification issues related to the number of parameter estimates.

### Model building in LTA

Before proposing our new framework of model building in LTA, we first review the methodological literature on different methods to fit LTA within the context of mixture modeling (Muthén and Shedden, 1999; Muthén and Muthén, 2000). In the area of model building related to LTA, recent methodologists have proposed a three-step method to study mixture models and to estimate the effects of covariates and distal outcomes in the model (Vermunt, 2010; Asparouhov and Muthén, 2014). This three-step method is defined as the following steps: fit a LCA using the categorical responses (Step 1), assign observations to latent classes based on the latent class posterior distributions, i.e., probabilities of being in each latent class (Step 2), and fit covariates and distal outcomes if available (Step 3). Asparouhov and Muthén (2014) showed the stability of latent class variables from the inclusion of covariates and distal outcomes and they also developed the standard errors for the Lanza method based on LCA in the three-step method that was not provided in Lanza et al. (2013). Nylund-Gibson et al. (2014) further explored the three-step method within a LTA framework describing a unique latent transition model where the measurement models are a latent class analysis (LCA) model and a growth mixture model by both modeling kindergarten readiness profiles and linking them to elementary students' reading trajectories. Although the three-step method described in Nylund-Gibson et al. (2014) is a “novel application” (p. 441) and also applicable to LTA consisting of cross-sectional LCAs, their example used only two time points of the one time before elementary school and the other time at elementary school. Thus, their method is not easily applicable to latent classes defined over more than two time points, although more than two time points are very common in longitudinal studies. Also, using different measures over time is not commonly employed in LTA. In this paper, we focus on a framework of model building in LTA using same measures over time.

On the other hand, there are some variations in model building in both LTA and mixture modeling when including covariates. For example, the three-step method in LTA discussed earlier (Vermunt, 2010; Asparouhov and Muthén, 2014) recommends to fit covariates and distal outcomes after determining latent statuses, whereas some of the mixture modeling literature suggests to consider covariates when determining latent profiles (Li and Hser, 2011). The discussion of the order of considering covariates is still controversial, but in our proposed new framework to fit LTA, we do not consider covariates when determining latent statuses because of the potential change between LTA models with and without covariates (Vermunt, 2010; Asparouhov and Muthén, 2015). However, if covariates are hypothetically supported, theoretically relevant, or shown to work in previous empirical studies, it is recommended to use covariates when enumerating latent statuses. In this paper, we extend the three-step method over three time points and propose a new framework of fitting LTA into longitudinal data in which latent classes are consistently defined over time.

### Empirical examples of inconsistency in model building using LTA

Applied researchers have focused on how to fit LTA to their data. For example, Miller et al. (2013) first used LCA to determine the class structure of the patterns of victimization and perpetrations (i.e., five groups with distinct profiles), and used gender and dummy codes for race/ethnicity (as covariates) to predict the classes in LCA. Then they estimated a LTA model to examine the transitioning from one class to another over time while constraining the latent classes to be equal across all three waves. Williford et al. (2014) fit LTA using LCA results without covariates to examine the patterns of bullying and victimization over time. Importantly, some studies have not applied a LCA before fitting a LTA (Goldweber et al., 2011; Castellini et al., 2013), which highlight the inconsistency in the LTA literature. Both strategies have their rationales. Specifically, the first strategy (fitting LTA using LCA) places more emphasis on the existence of heterogeneous groups that could be identified over time, whereas the later strategy places more emphasis on the stability of heterogeneous groups when the transition among latent statuses is considered. Readers will see later that both approaches were considered with the example of bullying data.

In summary, from the above examples, readers may have noticed the inconsistency in literature regarding fitting LTA. Such inconsistencies often occur when researchers try to reduce response categories, consider LCAs in model building of LTA, constrain parameters in LTA, and consider the order of modeling covariates and grouping variables. Some procedures in fitting LTA may not be unified, for example, the step of constraining parameters in LTA is often linked to a researcher's hypothesis and is content-specific. On the other hand, other consistent steps can be proposed in model building to provide applied researchers with a unified procedure for fitting LTA, which is one of the goals in the current paper. For example, regarding the inconsistency in whether to consider LCA in the model building in LTA, we think that if the changes in classes and their associations are expected, it is more reasonable to consider LTA with cross-sectional LCA. However, changes in the number of classes would be related to cross-sectional differences in the strengths of the presence of classes and the different cross-sectional associations of classes instead of the existence of a different number of classes over time. This means that it is more natural to consider the same number of classes over time and to understand the different number of classes as different strengths of the presence of classes and their associations instead of the changes of both the number of classes and their associations over time. The framework of fitting LTA we propose includes this assumption of equal number of latent statuses over time.

## A framework of fitting and evaluating LTA

**Figure 1:**
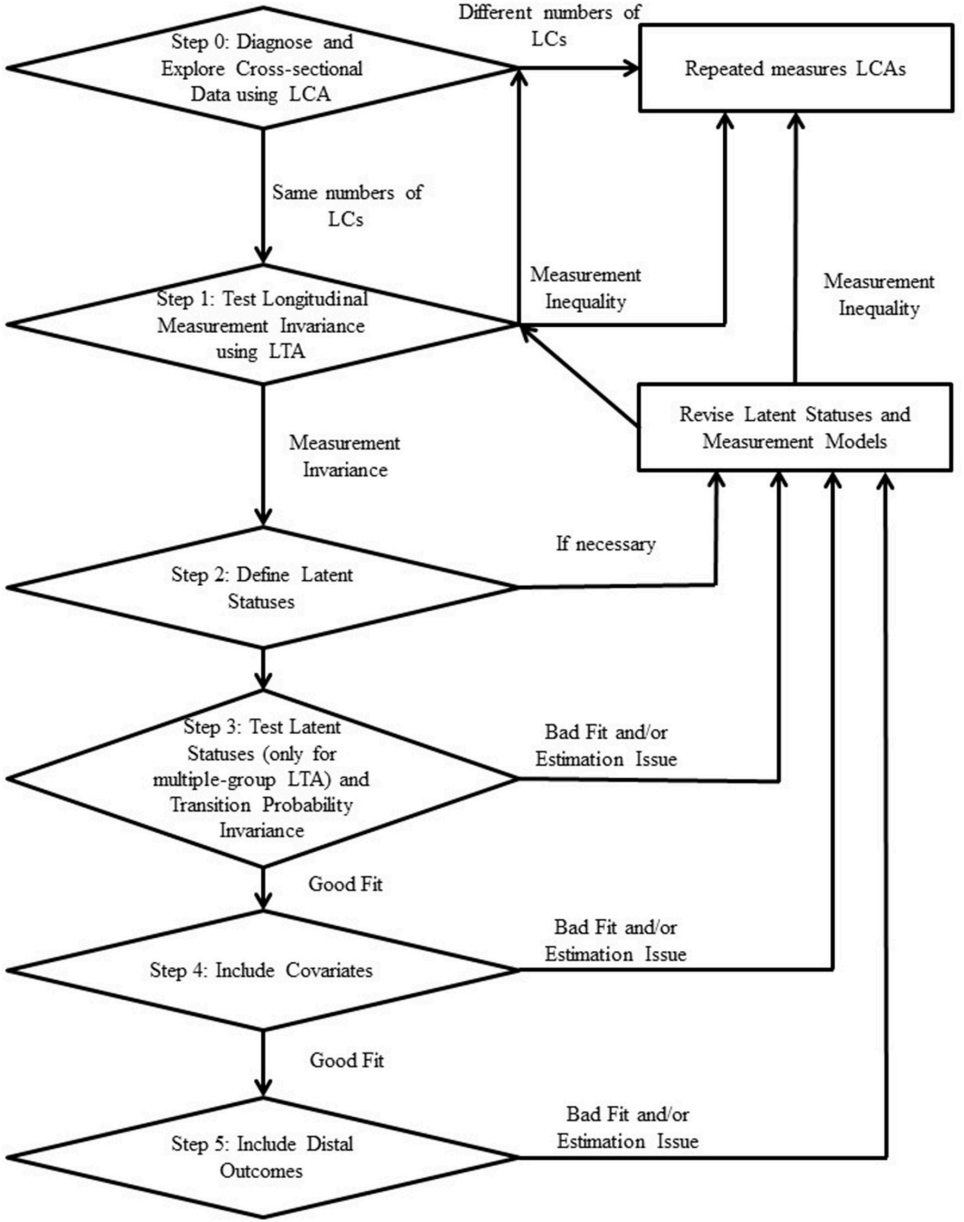
Flowchart of the procedure for model building using LTA.

Based on the review of existing literature (Collins and Lanza, 2010; Vermunt, 2010; Asparouhov and Muthén, 2014, 2015; Nylund-Gibson et al., 2014), we propose a synthesized framework of fitting LTA as illustrated in the flowchart in Figure 1 while considering possible grouping variables, the availability of covariates, and distal outcomes. The framework of fitting LTA synthesizes many different approaches used in empirical studies discussed earlier and provides a unified procedure of fitting LTA. The LTA model is classified as a model of both latent and observed categorical variables and the specific strategies to fit and evaluate LTA models are described in detail in Appendix A. Although latent categorical variables may resemble ordered categories, no order among latent statuses is necessarily assumed and latent statuses are nominal in scale. Rather, characteristics of latent statuses represented by item-response probabilities are interpreted as the resulting characteristics from a LTA.

Consistent with other longitudinal data analyses, it is very important and informative to diagnose and explore cross-sectional data first at each time point (Step 0). Although LTA is a latent variable model that can be considered as a special type of structural equation modeling (SEM), LTA does not necessarily adopt a confirmatory analysis, but rather is often applied as an exploratory analysis (Nylund-Gibson et al., 2014). That is, the number of latent statuses (or classes in LCA) is not a predetermined quantity. To explore the number of latent statuses, it is necessary to fit LCA into cross-sectional data over all of time points before running LTA. Aforementioned, considering covariates in enumerating latent classes depends on the role of covariates. That is, if covariates are known to play a key role in enumerating latent classes, it can be considered while enumerating latent classes in LCA. Otherwise, covariates should not be considered while enumerating latent classes due to the variability of latent classes. Considering our bullying data collected with the 6-item PRBm with three time points (*N* = 1,180), we examine three LCAs in three measurement times that provide different quantities for the number of latent classes and item-response probabilities. In this step, we create a pool of candidate models of latent statuses whose number of latent classes is the same over time and that are supported by at least one criterion of model selection (e.g., AIC or BIC as discussed in Appendix A.3). In the case that a researcher wants to have a different number of latent classes over time, it is recommended to fit a repeated measure LCAs by dropping the transition parts in LTA (Collins and Lanza, 2010). In other words, the researcher would fit a separate LCA for each of the cross-sectional data sets and then discuss how the observations form or merge classes over time. This technically would not be a transition analysis because one is not examining the movement of observations from one class to another, but rather how associations among the observations can change the number of classes and, subsequently, the qualitative description of the population over time.

The results obtained from the exploratory step (Step 0) may or may not be statistically meaningful in terms of measurement variances corresponding to item-response probabilities. In Step 1, longitudinal measurement invariance of item-response probabilities across times will be formally tested with the best choice of latent statuses, so that the latent statuses preserve their characteristics over time. The measurement invariance test will be conducted by model comparison between the measurement variance model and the measurement invariance model within the best candidate model. In the case of failure in longitudinal measurement invariance of item-response probabilities across times with the best candidate model, the next best candidate model will be applied. The procedure of model comparison is described in detail in Appendix A.3. If none of the candidate models with varying numbers of latent classes holds longitudinal measurement invariance of item-response probabilities across times, it is recommended to fit repeated measures of LCAs with latent classes inconsistently defined (Collins and Lanza, 2010).

When longitudinal measurement invariance is achieved, applied researchers are able to define (or name) the latent statuses that are consistently identified over time (Step 2). In step 2, it is recommended to compare different measurement invariant models constraining a subset of the item-response probabilities, to find a best fitting model in the studied sample. In the case that parameter constraints are meaningful in the sample, applied researchers are recommended to confirm if such constraints are statistically valid. If a model does not fit well both theoretically or empirically, it is recommended to go back and pick another candidate model with different latent statuses in Step 1 until the LTA model is an optimal model and then label them again in Step 2. In Step 3, if there are more than one latent status, we test the invariance of latent prevalence rates. We also test the transition probability invariance, if there are more than two time points. Again, the whole process is exploratory, and thus, it is recommended to choose, and test, other candidate models from the model candidate pool if the current model indicates unstable estimates or is not meaningful.

If additional covariates and/or distal outcomes are available, it is recommended to include covariates (Step 4) first and then distal outcomes (Step 5) on the LTA model selected in Step 3. In the following sections, we will describe the framework of fitting LTA using the data on students' concerns about bullying. The data and command (syntax) files needed for reproducing the results using SAS PROC LCA and PROC LTA (Version 1.3.2) (2015) and M*plus* 7.4 (Muthén and Muthén, 1998–2015) are provided as online supplementary materials. Exemplary syntaxes of SAS Proc LCA/LTA and Mplus were added in Appendix B.

## Empirical example: student-centered concerns about bullying

### Introduction

Middle school years and the transition into and out of middle school present an important period to study bullying. Interestingly, as bullying increases between elementary school and middle school, students' attitudes toward bullying and aggression become more positive during middle school years compared to elementary school years (Oliver et al., 1994; Graham and Juvonen, 1998; Pellegrini and Bartini, 2000). In addition, students are more attracted to aggressive peers during middle school years (Bukowski et al., 2000). Adolescents who were aggressive during middle school were rated as popular by their non-aggressive peers, and were identified as the nuclear members of the social network (Cairns et al., 1988). Students who were nominated as bullies were significantly less isolated compared to all the other peers including victims, bully-victims, and uninvolved students (Veenstra et al., 2005). Taken together, young adolescents might affiliate with aggressive peers or tolerate bullying behaviors in order to “explore new social roles and challenge adult-endorsed social norms” (Pellegrini, 2002, p. 152), which provides an environment that supports the increase in aggressive behaviors during the middle school years.

Although students' attitudes toward bullying become more positive during middle school, studies have shown that both elementary and middle school students were concerned about school safety and bullying (Astor et al., 2001; Robers et al., 2013). Studies show that students report that they are still worried about bullying at school, ranging from 19 to 40% (Akos, 2002; Jones, 2004), including 27% of 3rd to 5th graders worrying about cyber bullying (D'Antona et al., 2010). The concerns and fear about school bullying contributes to feeling unsafe for many students, regardless of race (Bachman et al., 2011b). As a result of concerns of bullying and school safety, some students end up avoiding going to school, not attending certain courses, and avoiding after school activities (Randa and Wilcox, 2010). Furthermore, some research has found that the effect of concerns about bullying varied for students in different grades. For example, Hughes et al. (2015) found that feeling unsafe due to bullying at school was a significant predictor for students missing school for 12th graders. On the other hand, concerns about cyber bullying were a significant predictor for 9th graders missing school due to feeling unsafe. Research has also found gender and grade level differences. Specifically, less school bullying contributed to feelings of safety for male and female 5th graders, and for 8th grade girls, but not for 11th graders (Bachman et al., 2011a). Consistent with their concerns about bullying, many students (65%) do not think their schools are doing a good job in dealing with bullying (Marley, 2008). Students' pervasive concerns about bullying, their dissatisfaction with school-based bullying interventions, and the detrimental outcomes associated with those concerns (e.g., school avoidance) highlight the importance of studying students' concerns about bullying over time, including the impact of gender and grade level on the changes in bullying concerns.

Most studies examining the changes in students' concerns about bullying and prevalence rates have used cross-sectional designs. Based on our knowledge, no studies have used longitudinal designs to examine the changes in students' concerns about bullying over time, which may result in misunderstanding the changes in students' concerns over time.

The current study employed LTA to estimate the prevalence of bullying concerns and degree of transitioning between latent statuses over time. Instead of directly defining students' concerns about bullying using an observational measure, LTA allows us to classify heterogeneous subgroups (or latent classes) for students' concerns about bullying. This example seeks to address the following questions:

1. Are there qualitatively distinct subgroups of students who demonstrate particular patterns of concerns regarding bullying?
2. What is the probability that an individual will be in a different latent status?
3. Is there change between latent statuses across time? If so, how can this change be characterized?
  - If an individual is in a particular latent status at Time *t*, what is the probability that the individual will be in that latent status at Time *t*+1?
4. Is the change in latent group status affected by a student's grade (using LTA with a covariate)?

### Method

#### Participants

Participants at Time 1 were 1,180 students ranging from 5th to 9th grade attending nine schools in a mid-western city in the United States, with university and school district IRB approval. All students were given consent forms to take home to their parents, which explained the nature of the study. Almost all student (97%) gave assent to participate the study. Due to students' school transitions, the number of schools was expanded to 22 over three semesters. The mean age was 12.2 years (*SD* = 1.29 years) with 9.9% indicating that English was not their first language. Grades were distributed as follows: 5th (10.0%), 6th (31.4%), 7th (26.4%), 8th (21.0%), and 9th grade (10.6%) at Time 1. The assessments were administered over three semesters. The attrition rates were 5.59% at Time 2 and 15.34% at Time 3. This sample has been used in previous studies for different research questions, but the specific variables used in this study have not been previously analyzed (Swearer et al., 2012; Ryoo et al., 2015). Readers can refer to a previous paper (Ryoo et al., 2015) for detailed procedure of the study.

#### Measures

Each student completed a demographic questionnaire that included questions about gender, age, grade, first language use, and race/ethnicity. Then, students completed the Pacific-Rim Bullying measure (PRBm; Konishi et al., 2009), which surveyed students' experiences and concerns about bullying and victimization. Data were also collected from school records that included demographics, students' cognitive assessment scores, GPA, and office referral data.

#### Concerns about bullying

Students answered six questions about their concerns about being the target of bullying on a four-point Likert-type format (“*No, not at all,” “No, not much,” “Yes, a little,” and “Yes, very much,”* see column 1 in Table 1). Instead of using all four response options, two options were used by collapsing the first two categories together (“*No”*) and by merging the last two categories together (“*Yes”*). Sample internal consistency of reliability (α) estimates for the concerns about bullying scale were 0.79 (Time 1), 0.79 (Time 2), and 0.80 (Time 3). Table 1 summarizes the proportions and items in detail and shows that the changes of marginal proportions are not consistent across the items. However, such inconsistences may not be interpretable because they are not inferential statistics, but descriptive statistics. In applying LTA, we will derive the inferential results that allow us to generalize our findings beyond our sample to the larger population of students from this particular city.

**Table 1:** Marginal response percentages^a^ for items indicating concerns about bullying in PRBm^b^.

**How concerned or afraid are you that you might …**	**Time 1**		**Time 2**		**Time 3**	
	**No**	**Yes**	**No**	**Yes**	**No**	**Yes**
Be physically attached or hurt by another student or a group of students.	80	20	82	18	86	14
Have other students talk you into doing things that you are not comfortable with.	77	23	82	18	85	15
Be made fun of or left out because of your culture or race.	88	12	89	11	91	9
Be made fun of or left out because of your beliefs or ideas.	82	18	82	18	84	16
Have rumors or gossip spread about you.	67	33	66	35	74	27
Be verbally harassed or embarrassed at school.	74	26	76	24	79	21

a *Percentages were multiplied by 100*.

b *PRBm is acronym for Pacific-Rim Bullying measure*.

### Results

To address the empirical example research questions, we fit LTA to the students' concerns about bullying data by following the proposed framework as discussed in the previous section. All analyses and results presented herein were based on SAS LCA/LTA (2015). The data and command (syntax) files needed for reproducing the results using SAS LCA/LTA (2015) and M*plus* 7.4 (Muthén and Muthén, 1998–2015) are provided in the Supplementary Online materials in the main folders labeled SAS and M*plus* with corresponding subfolders labeled for each step in the analysis process (i.e., Step 0, … Step 4).

**Step 0: Diagnose and Explore Cross-sectional Data using LCA**. As shown in Table 1, the probability of students having bullying concerns varied across specific bullying items as well as over time. In this step, it is necessary to cluster responses into several homogeneous groups at each time point using LCA so that we can characterize the heterogeneous groups. Table 2 summarizes the results for the LCAs, which show that the four-solution and the three-solution models were favored with respect to fit indices of AIC, BIC, CAIC, and ABIC being the lowest. Although the entropy measuring certainty in classifying latent statuses favored a two-solution model, we did not consider the two-solution model because simple high/low separation did not provide greater descriptions of students' concerns about bullying than factor analysis. Next, we created a pool of candidate models with three or four latent statuses. As previously stated in the section titled “Literature Review on Model Selection in LTA,” we considered the same number of latent statuses over time and thus, the pool included only the three-solution model and four-solution model.

**Table 2:** Results of LCA at each time point (Step 0).

	***G*^2^^a^**	**AIC^b^**	**BIC^c^**	**CAIC^d^**	**ABIC^e^**	**Entropy**	**DF^f^**
**Time 1**							
2-solution	191.98	217.98	283.84	296.84	242.55	**0.81**	50
3-solution	53.52	93.52	**194.85**	**214.85**	**131.32**	0.79	43
4-solution	35.00	**89.00**	225.80	252.80	140.04	0.78	36
5-solution	23.54	91.54	263.80	297.80	155.80	0.76	29
6-solution	20.43	102.43	310.16	351.16	179.93	0.77	22
7-solution	17.27	113.27	356.46	404.46	203.99	0.67	15
**Time 2**							
2-solution	161.38	187.38	252.57	265.57	211.27	**0.84**	50
3-solution	59.24	99.24	**199.52**	**219.52**	**135.99**	0.82	43
4-solution	41.90	**95.90**	231.28	258.28	145.52	0.80	36
5-solution	30.36	98.36	268.84	302.84	160.84	**0.84**	29
6-solution	23.36	105.36	310.93	351.93	180.71	0.82	22
7-solution	18.84	114.84	355.50	403.50	203.04	0.82	15
**Time 3**							
2-solution	187.65	213.65	277.37	290.37	236.09	**0.85**	50
3-solution	75.25	115.25	**213.29**	**233.29**	**149.76**	0.84	43
4-solution	54.00	**108.00**	240.35	267.35	154.60	0.84	36
5-solution	41.84	109.84	276.50	310.50	168.51	0.82	29
6-solution	28.15	110.15	311.12	352.12	180.91	0.81	22
7-solution	21.40	117.40	352.68	400.68	200.23	0.76	15

*Bold indicates the best solution model for the corresponding fit index*.

a *G^2^ is the likelihood ratio statistics*.

b *AIC is Akaike Information Criterion*.

c *BIC is Bayesian Information Criterion*.

d *CAIC is consistent AIC*.

e *ABIC is adjusted BIC*.

f *DF is degree of freedom*.

**Step 1: Test Longitudinal Measurement Invariance using LTA**. In the previous step, the item response probabilities were not constrained and thus, may cause ambiguity when defining latent statuses at Step 2 because the characteristics of latent statuses can be explained by observed items as well as variance of the measurement model. The goal of testing longitudinal measurement invariance is to achieve measurement invariance, so that the characteristics of latent statuses can be explained by observed items over time. Results showed that both the three-solution model and four-solution model indicated that measurement invariances hold from the likelihood ratio difference test (LRDT; $G_{\Delta }^{2}=49.74$, *df*_Δ_ = 36, *p* > 0.05 and $G_{\Delta }^{2}=56.24$, *df*_Δ_ = 48, *p* > 0.05 for three-solution and four-solution models, respectively), AIC, and BIC (see Table 3).

**Table 3:** Results of longitudinal measurement invariance (Step 1).

	**MI**	***G*^2^^a^**	**AIC^b^**	**BIC^c^**	**DF^d^**	**Diff.*G*^2^^e^**	**Diff.DF^f^**	***P*-value**
3-solution	Yes	3824.65	**3888.65**	**4050.99**	262111			
	No	3774.91	3910.91	4255.89	262075	49.74	36	0.063492
4-solution	Yes	3701.09	**3803.09**	**4061.83**	262092			
	No	3644.85	3842.85	4345.11	262044	56.24	48	0.193694

*Bold indicates the best solution model for the corresponding fit index*.

a *G^2^ is the likelihood ratio statistics*.

b *AIC is Akaike Information Criterion*.

c *BIC is Bayesian Information Criterion*.

d *DF is degree of freedom*.

e *Diff.G^2^ is the difference of likelihood ratio statistics*.

f *Diff.DF is the difference of degrees of freedom*.

**Figure 2:**
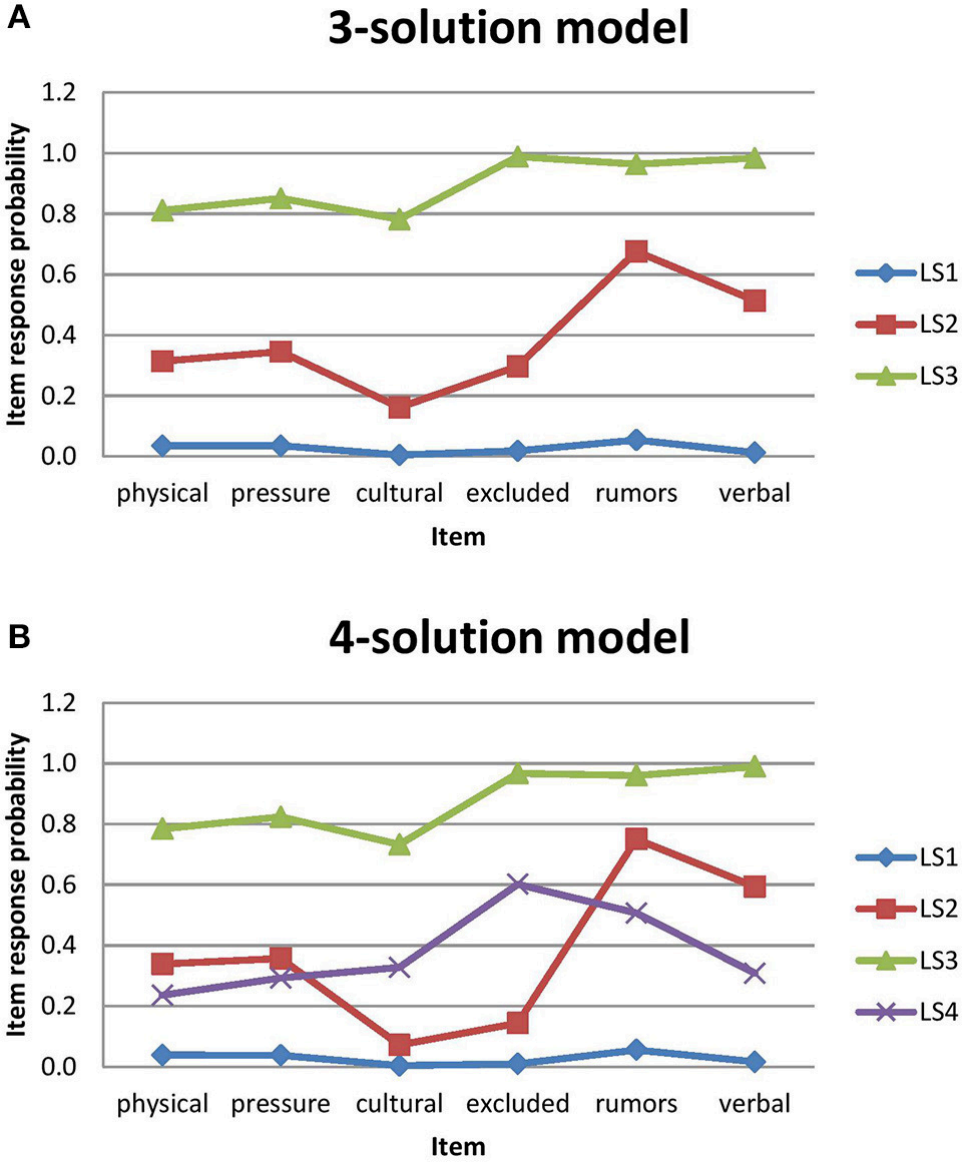
Latent statuses profiles for three-solution and four-solution models. Physical = concerned or afraid that you might be physically attached or hurt by another student or group of students; Talking (pressure) = concerned or afraid that you might have other students talk you into doing things that you are not comfortable with; Cultural = concerned or afraid that you might be made fun of or left out because of your culture or race; Belief (excluded) = concerned or afraid that you might be made fun of or left out because of your beliefs or ideas; Spread (rumors) = concerned or afraid that you might have rumors or gossip spread about you; Verbal = concerned or afraid that you might be verbally harassed or embarrassed at school. **(A)** 3-solution model. **(B)** 4-solution model.

**Step 2: Define Latent Statuses**. Steps 0 and 1 encompassed three-solution and four-solution models with measurement invariance as candidate models. Based on the item-response probabilities within each latent status, we plotted profiles of three-solution and four-solution models in Figure 2. We selected a four-solution model from the pool of candidate models that holds measurement invariance because AIC and BIC are lower (see Table 3). Also, all of the four latent statuses represent meaningful groups regarding students' concerns about bullying. We realize that other researchers, examining these fit statistics, could reasonably argue that the more parsimonious three-solution model should be preferred. However, model parsimony is not necessarily the goal of LTA. The goal of LTA, particularly when it is used in an exploratory vein, is to describe the data by identifying classes within the population of observations in the data and the selection of the four-solution model provides a more nuanced description of the data. Additionally, when we examined which classes were being combined when moving from a four class model to a three class model, we saw that the two classes at the center of the overall data distribution were being combined while the classes at the extremes of the overall data distribution remained almost unchanged in Figure 2. This is not an unusual finding because the posterior probabilities used to assign observations to classes at the center of the overall data distribution are likely to be less clear (i.e., closer to 0.5) than the probabilities at the extremes of the distribution. Further, the sample for our exploratory analysis comes from a single city in the Midwest of the United States. Given that all of the observations come from a single geographic region, it is likely that the associations among these observations are likely to be greater than would be expected under the assumption of simple random sampling. With a more diverse sample the separation between the two center classes may become more distinct. Moreover, it is important to note that fit statistics are not strict statistical tests, but rather guidelines to assist researchers in determining the correct number of latent classes. Fit statistics are meant to be used in conjunction with theoretical considerations. Since the fit statistics lend support to the four-solution model, each of the classes in the four-solution model is theoretically meaningful, the goal of this study is exploratory, and there is a potential of confounding associations within the data, we deemed that the four-solution model was the preferred model to move forward in the analysis. This enumeration addressed the first research question: Are there qualitatively distinct subgroups of students who demonstrate particular patterns of concerns regarding bullying?

Based on the item probabilities, we could classify the four different groups for student-centered concerns about bullying as: *not concerned* (LS1) referred to the unconcerned students; *rumor-concerned* (LS2) referred to the students who are concerned about rumors or gossip (0.75) and being verbally harassed or embarrassed (0.59); *social exclusion-concerned* (LS3) referred to the students who are concerned about being made fun of or being left out because of their beliefs/ideas (0.60) or culture/race (0.33), rumors or gossip (0.51); and the *most concerned* (LS4) referred to the students who are very concerned about all six forms of bullying with high probabilities (>0.73) (see Table 4).

**Table 4:** Probabilities of item parameters (ρ estimates) on student's concerns about bullying (Step 2).

**Latent status**		**LS1**	**LS2**	**LS3**	**LS4**
**Response category**		**Yes**			
All	Physical	0.0380	0.3386	**0.7844**	0.2357
	Talking	0.0375	0.3568	**0.8232**	0.2919
	Cultural	0.0035	0.0715	**0.7326**	0.3276
	Belief	0.0087	0.1437	**0.9664**	**0.6014**
	Spread	0.0549	**0.7499**	**0.9597**	**0.5069**
	Verbal	0.0156	**0.5932**	**0.9890**	0.3083

*LS stands for latent status; LS1 - Not concerned; LS2 - Rumor-concerned; LS3 - Most Concerned; LS4—social exclusion-concerned. Physical, concerned or afraid that you might be physically attached or hurt by another student or a group of students; Talking, concerned or afraid that you might have other students talk you into doing things that you are not comfortable with; Cultural, concerned or afraid that you might be made fun of or left out because of your culture or race; Belief, concerned or afraid that you might be made fun of or left out because of your beliefs or ideas; Spread, concerned or afraid that you might have rumors or gossip spread about you; Verbal, concerned or afraid that you might be verbally harassed or embarrassed at school. Bold indicates the probability > 0.50*.

Table 5 summarizes the latent status prevalence and the transitions over the three time points for students concerns about bullying. Starting with the δ estimates (first block of Table 5) we can examine prevalence rates over the three time points. Next, the second block of Table 5 summarizes the transition matrix from Time 1 (row) to Time 2 (column) and the third block of Table 5 summarizes the transition matrix from Time 2 (row) to Time 3 (column).

**Table 5:** Latent status prevalence (δ estimate) and transition matrix estimates (τ estimates) over three time points on student's concerns about bullying.

		δ **estimate**					τ **estimate^a^**					τ **estimate^b^**			
	**Time**	**LS1**	**LS2**	**LS3**	**LS4**		**LS1**	**LS2**	**LS3**	**LS4**		**LS1**	**LS2**	**LS3**	**LS4**
All	Time1	0.5691	0.2395	0.0804	0.1109	LS1	**0.8252**	0.0925	0.0208	0.0615	LS1	**0.8518**	0.0703	0.0143	0.0637
	Time2	0.5961	0.2166	0.0719	0.1155	LS2	**0.2780**	**0.5734**	0.0893	0.0594	LS2	**0.3666**	**0.5473**	0.0861	0.0000
	Time3	0.6695	0.1625	0.0628	0.1052	LS3	**0.2707**	**0.3309**	**0.3785**	0.0198	LS3	**0.2639**	0.0287	**0.4959**	**0.2114**
						LS4	**0.3432**	0.0000	0.0739	**0.5829**	LS4	**0.5492**	0.0000	0.0000	**0.4508**

*LS, stands for latent status; LS1, not concerned; LS2, rumor-concerned; LS3, most concerned; LS4, social exclusion-concerned. Bold indicates the probability > 0.20*.

a *Transition matrix from Time 1 to Time 2*.

b *Transition matrix from Time 2 to Time 3*.

The prevalence rates indicated that the probability of being in the *rumor-concerned* (LS2) group was relatively high; 0.24 at Time 1, 0.22 at Time 2, and 0.16 at Time 3, whereas the probabilities of being in the *social exclusion-concerned* (LS4) group were 0.11 at Time 1, 0.12 at Time 2, and 0.11 at Time 3. Also, the prevalence rates indicated that the probabilities of being in the *most concerned* (LS3) group were relatively low, 0.08 at Time 1, 0.07 at Time 2, and 0.06 at Time 3 whereas the probabilities of being in the *not concerned* (LS1) group were the highest among all four groups, 0.57 at Time 1, 0.60 at Time 2, 0.67 at Time 3. This latent status prevalence addressed the second research question: What is the probability that an individual will be in a different latent status?

We found that the *most concerned* (LS3) group tended to move into either the *rumor-concerned* (LS2) from Time 1 to Time 2 ($\tau_{2|1}^{LS3\to LS2}=$ 0.33) or the *social exclusion-concerned* (LS4) from Time 2 to Time 3 ($\tau_{3|2}^{LS3\to LS4}=$ 0.21) or stayed within the *most concerned* (LS3) group ($\tau_{2|1}^{LS3\to LS3}=$ 0.38 or $\tau_{3|2}^{LS3\to LS3}=$ 0.50). All of *concerned* groups tended to move into the *not concerned* or stayed within each group.

**Step 3: Test Latent Statuses (only for multiple-group LTA) and Transition Probability Invariance**. As indicated in Table A.1 in Appendix A, transition probability invariance should be tested, to confirm its necessity. We formally tested the transition probability invariance with the four-solution model and summarized the results in Table 6. The results indicated that AIC and BIC favored the transition probability invariant model while the LRDT ($G_{\Delta }^{2}=21.84$, *df*_Δ_ = 12, *p* < 0.05) favored the free transition probability model (see Table 6). Although the similarity between transition matrices (τ_2|1_ and τ_3|2_) was observed in Table 5, we found large and meaningful probabilities in the transition matrices suggesting different transition patterns at different time points. For example, different transition estimates from the *most concerned* (LS3) group to the *rumor-concerned* (LS2) group at two different time points (0.33 vs. 0.03) and from the *most concerned* (LS3) group to the *social exclusion-concerned* (LS4) group (0.02 vs. 0.21) (see Table 5). Thus, we chose the four-solution model with free transition probabilities. This confirmation of transition matrices addressed the third research question: Is there change between latent statuses across time? If so, how can this change be characterized?

**Table 6:** Result of transition probability invariance (Step 3).

**4-solution**	***G*^2^^a^**	**AIC^b^**	**BIC^c^**	**DF^d^**	**Diff.*G*^2^^e^**	**Diff.DF^f^**	***p***
Model 2 (invariance)	3722.93	**3800.93**	**3998.79**	262104			
Model 1	3701.09	3803.09	4061.83	262092	21.84	12	0.039

*Bold indicates the best solution model for the corresponding fit index*.

a *G^2^ is the likelihood ratio statistics*.

b *AIC is Akaike Information Criterion*.

c *BIC is Bayesian Information Criterion*.

d *DF is degree of freedom*.

e *Diff.G^2^ is the difference of likelihood ratio statistics*.

f *Diff.DF is the difference of degrees of freedom*.

**Step 4: LTA with Covariates**. The variable, grade, was added to the four-solution model with free transition probabilities as a covariate. We found significant effects of grade on the prevalences ($G_{\Delta }^{2}=8.88$, *df*_Δ_ = 3, *p* < 0.05), but could not obtain any results on the effect of grade on the transition probabilities because an estimation problem occurred due to the sparseness. The result of the effect of grade was summarized in Table 7. Compared with the *not concerned* (LS1) group, the odds ratios for the *rumor concerned* (LS2) group and the *most concerned* (LS3) group are less than one (0.9786 for LS2; 0.7289 for LS3) while the odds ratio for the *social-exclusion concerned* (LS4) is greater than one (1.0801). Thus, as students get older, they are less likely to be in LS2 or LS3 but more likely to be in LS4 than in the *not concerned* (LS1) group. For example, the odds of being in the most concerned (LS3) group for 6th graders student was 0.7289 times the odds of being the most concerned (LS3) group for 5th graders.

**Table 7:** Effect of grade on the latent prevalences (Step 4).

	**Time 1 Latent Status**			
	**Not concerned**	**Rumor-concerned**	**Most concerned**	**Social-exclusion concerned**
**INTERCEPT**				
β_0_'s	Reference	−0.7767	−1.0814	−1.9299
Odds	Reference	0.4599	0.3391	0.1452
**Grade**				
β_1_'s	Reference	−0.0216	−0.3162	0.0771
Odds	Reference	0.9786	0.7289	1.0801

*β_0_ and β_1_ are regression coefficients for logistic regression models*.

It should be noted that the same problem, the effect of grade on the prevalences was testable while the effect of grade on the transition probabilities caused estimation error, occurred when we tested a three-solution model. The sparseness here is not due to the use of a four-solution model as opposed to a three-solution model because, as noted above, the two larger classes of the four-solution model are the ones that are combined together in the three-solution model. This investigation of the effect of covariates addressed the fourth research question: Is the change in latent group status affected by a student's grade (using LTA with a covariate)? In this study, we did not consider any distal outcomes (Step 5 in Figure 1).

### Discussion for empirical example

Few studies have explored the changes in student-centered concerns about bullying over time. Based on the current results, we found four distinct types of concerns over three time points from late elementary to high school. In general, students who are concerned about bullying are more likely to change statuses over time compared to students who are not concerned about bullying, suggesting that concerns co-vary across types of bullying behaviors.

#### Prevalence rates

In the current study, we found that adolescents were concerned about bullying, especially about relational bullying (rumor, verbal harassment, and social exclusion). Specifically, 33.0% to 43.1% of adolescents expressed concerns about different types of bullying over time. This supports findings from previous studies that middle school students were concerned about bullying (Akos, 2002; Jones, 2004; D'Antona et al., 2010). Specifically, we identified four distinct groups of students: *most-concerned group, rumor-concerned, social exclusion-concerned* and *not concerned group*. We found that students' concerns about bullying somewhat mirrored the national bullying prevalence data, specifically, the higher prevalence of verbal bullying (17.6%) and rumors (18.3%), and relatively lower prevalence of physical bullying (7.9%) among adolescents (Robers et al., 2013). Instead of being exclusively concerned about physical types of bullying, the most common profiles of students' concerns of bullying included an emphasis on verbal/relational bullying (e.g., rumors), and social exclusion (e.g., being made fun of or left out). This finding suggests the importance of intervening during verbal/relational bullying and social exclusion, instead of only focusing on physical bullying, which is the focus of bullying prevention in many schools. Because concerns about bullying often lead to other behavioral and psychosocial difficulties, such as school avoidance, and because few studies have examined students' concerns about bullying over time, future applied researchers need to recognize that bullying is a complex behavior that should be examined longitudinally. Also, bullying encompasses several forms: verbal, relational, physical, and electronic. When possible, applied researchers should study all forms simultaneously.

#### Changes in concerns about bullying

Students' concerns about bullying also decreased over time and narrowed to specific concerns about rumors, gossips and social exclusion. Furthermore, older students were less likely to be in the *rumor-concerned* and *most concerned* groups compared with being in the *not concerned* group, but more likely to be in the *social exclusion-concerned* group. This is consistent with the literature that bullying takes more subtle forms (vs. physical forms) as students get older (Coie and Dodge, 1998). However, it is not clear if the decrease in students' general concerns about bullying is due to the decrease in the prevalence of bullying (especially physical bullying) over time, or to students' mastery of new strategies to cope with bullying. Future studies should examine whether changes in students' concerns about bullying are related to other individual factors such as coping strategies.

#### Limitations and future directions

There are several limitations in the current study. First, limited conclusions about causal–effect relations can be drawn from the results. Furthermore, the students in the current study were recruited from nine schools in one city in the Midwest and most students were European Americans. The findings from this study may not be readily generalizable to students living in rural areas or other socially and politically different areas because students' experiences with bullying are likely to differ depending on the location of the schools (e.g., inner city vs. rural areas, geographic region).

In addition, we were not able to examine the effect of grade on the transition probabilities because an estimation problem occurred due to the sparseness. The estimation problems were not related to the selection of four latent statuses instead of three-solution model because the same estimation problem occurred with three-solution model. Rather, the *social exclusion-concerned* group held similar characteristics with the *rumor-concerned* group in the physical and peer pressure groups and these two groups were clustered in the three-solution model. Thus, the *most concerned* group with prevalence rates less than 10% did not seem to be related to the increase of latent statuses. Therefore, requiring a relatively large sample size and a subgroup size for more complex latent transition model would reduce such limitation of the LTA approach. Furthermore, the taxonomy of categorical and continuous latent variables was not discussed because it is beyond the scope of this study. However, it is necessary for applied researchers to explore if either a categorical or a continuous latent construct is fitted to the given data. Regardless, the proposed procedure of fitting LTA still includes subjective model building in the case of disagreement of the model fit evaluation. Future studies should use a larger sample of participants in different grades and from diverse backgrounds to continue examining changes in students' concerns about bullying and the contributing factors.

#### Implications

Most students in this sample expressed concerns regarding bullying, especially relational bullying. In addition to implementing specific interventions for students who frequently bully others, it is extremely important to encourage the majority of the students (bystanders) to speak up when bullying occurs (Polanin et al., 2012). Because students were more concerned about verbal and relational bullying than physical bullying over time, it is also important for adults to take relational bullying seriously and intervene not only during physical bullying, but also during relational bullying and social exclusion. In order to prevent bullying in all American schools, bullying interventions need to include all students and staff, address all forms of bullying, and be developmentally-based, gender- and culturally-sensitive, and responsive to all students' concerns.

## Conclusion

Our manuscript makes both a theoretical contribution about student-centered concerns about bullying and a methodological contribution regarding model building in LTA. Specifically, we found that the most common profiles of students' concerns of bullying included an emphasis on verbal/relational bullying (e.g., rumors), and social exclusion, and that there was no profile detected that endorsed physical bullying concerns without endorsing other types of bullying. In addition, students' concerns decreased over time and narrowed to specific concerns about rumors, gossips, and social exclusion over time.

### Limitation of the proposed model building framework

First, the proposed framework is limited to LTA model building for cases of the equal number of latent statuses over time. For cases of unequal number of latent statuses or hybrid models with growth mixture modeling (Masyn, 2013; Nylund-Gibson et al., 2014), this framework may not work well without any modifications. Second, this framework does not consider a new three-step method dealing with a measurement parameter shift problem that fixes the parameters estimated based on an unconditional model when adding covariates and/or distal variables (Vermunt, 2010; Asparouhov and Muthén, 2014). Although the new three-step method has been shown to be less biased, has lower mean squared error, and good confidence interval coverage, the conditions were somewhat limited. For example, Asparouhov and Muthén (2014) considered only two sample sizes of 500 and 2,000. Thus, more research is still needed for the three-step method to be generalizable. Finally, this paper does not apply additional model evaluation tools such as Lo-Mendell-Rubin (LMR) test or the bootstrap likelihood ratio test (BLRT) by focusing on the model building procedure. Further fit indices can be found at Nylund et al. (2007) and they can be replaced with AIC and BIC in the proposed framework of model building in LTA. However, as noted in Appendix A, the effectiveness of LMR and BLRT has not been studied in LTA.

Model building in LTA has not been fully discussed in the literature but the importance and applicability have been emphasized when the latent statuses make sense within a specific research study. The current study proposes a synthesized framework of fitting LTA in an exploratory fashion so that applied researchers can apply the method in their studies. Specifically, the flow chart described in Figure 1 encompasses the scattered model building procedures and synthesizes the method used in many empirical studies discussed in the introduction. In the literature in model building in LTA, there are still many issues including fitting distal outcomes without attenuated estimates that were often observed in the LCA literature. Nevertheless, this framework provides a unified tool in model building using LTA on data regarding student-centered concerns about bullying. When researchers identify the complexity of different factors (e.g., grade, concerns about bullying, developmental changes) that underlie the bullying dynamic, interventions can be developed and tailored to address these complexities across important developmental contexts with the goal of ending bullying among school-aged youth.

## Ethics statement

All procedures performed in studies involving human participants were in accordance with the ethical standards of the institutional and/or national research committee and with the 1964 Helsinki declaration and its later amendments or comparable ethical standards.

## Author contributions

JR: designed the study, performed the statistical analysis, and drafted the manuscript; CW and SS: participated in designing the study, collected data, and drafted the manuscript; MH: participated in drafting the manuscript; DS: conducted the literature review. All authors read and approved the final manuscript.

### Conflict of interest statement

The authors declare that the research was conducted in the absence of any commercial or financial relationships that could be construed as a potential conflict of interest.
