# Supplementary material for: Longitudinal Model Building Using Latent Transition Analysis: An Example Using School Bullying Data
Source: Front Psychol. 2018 May 8;9:675. doi: 10.3389/fpsyg.2018.00675 (PMC5953336; doi:10.3389/fpsyg.2018.00675)
Supplement: Supplementary file 1 [file Data_Sheet_1.docx]

**Appendix A. How to Fit and Evaluate LTA**

To better understand the framework of fitting LTA, in this appendix, we discuss model equations of LCA and LTA, parameter estimates, and model comparison tools in LTA. All of components and evaluation tools are discussed within the model selection procedure of LTA.

**A.1 Model Equations**

**Latent Class Analysis**. LTA can be conducted to represent the complex array of marginal probabilities in a format that is more parsimonious and easier to comprehend, and at the same time to reveal unobserved heterogeneous groups and their membership changes represented by the data. To better understand LTA, we briefly introduce the cross-section version of LTA, latent class analysis (LCA). Using the same notation as that in Collins and Lanza (2010), the LCA model can be written as:

$$P(Y=y)=\sum_{k=1}^{K} \delta_{k}\prod_{j=1}^{J} \prod_{r_{j}=1}^{R_{j}} \rho_{(j,r_{j}|k)}^{I(y_{j}=r_{j})}$$

where $k=1,\cdots,K$ are the number of latent classes, $j=1,\cdots,J$ are observed variables having $r_{j}=1,\cdots,R_{j}$ response categories ($R_{j}$ can vary across item), $\delta_{k}$ is probability of membership in the $k^{th}$ latent class, $\rho$ is the item-response probabilities, and $I$ is a binary indicator function for $j^{th}$ item. Thus, parameters estimated in LCA are both $\delta_{k}$ and $\rho$, which allows the computation of degrees of freedom with a corresponding contingency table.

The most important step in the model selection in LCA is to determine the number ($K$) of latent classes. Based on the selected number (or possible number) of latent classes and the assumption of local independence, we uniquely define latent classes by examining the item-response probabilities ($\rho$). The primary result from LCA would be the probability of membership ($\delta_{k}$).

**Latent Transition Analysis**. The transition probabilities among latent classes can be explored using LTA. The transition probabilities describe the different patterns of movements among individuals characterized in the previous time point. Using the same notations as for LCA, the LTA with three time lags in the current study of students’ concerns about bullying can be written as:

$$P(Y=y)=\sum_{k_{1}=1}^{K} \sum_{k_{2}=1}^{K} \sum_{k_{3}=1}^{K} \delta_{k_{1}}\tau_{(k_{2}|k_{1})}\tau_{(k_{3}|k_{2})}\prod_{t=1}^{3} \prod_{j=1}^{J} \prod_{r_{j,t}=1}^{R_{j}} \rho_{(j,r_{j,t}|k_{t})}^{I(y_{j,t}=r_{j,t})}$$

where $\tau_{(k_{2}|k_{1})}$ represents the probability of a transition to latent status $k$ at time 2, conditional on membership in latent status $k$ at time 1 and $\tau_{(k_{3}|k_{2})}$ represents the probability of a transition to latent status $k$ at time 3, conditional on membership in latent status $k$ at time 2.

Similar to the item-response probabilities ($\rho$) and the probability of membership ($\delta_{k}$), the transition probabilities ($\tau$) can also be tested to see if the transitions are consistent over time. In practice, applied researchers often restrict parameters of item-response probabilities, $\rho$, across times, i.e., $\rho_{(j,r_{j,t}|k_{t})}^{I(y_{j,t}=r_{j,t})}=\rho_{(j,r_{j}|k_{t})}^{I(y_{j,t}=r_{j})}$. The purposes of assuming measurement invariance across times and latent statuses are the same as in Collins and Lanza (2010), which are to easily interpret the latent transition models, to help stabilize estimation, and to improve identification.

However, instead of assuming measurement invariance, we recommend that applied researchers test measurement invariance because of the parameter drift on item-response probability over time. Testing measurement invariance over time is necessary to interpret the characteristics of latent statuses consistently. In the current study of students’ concerns about bullying, we formally tested the measurement invariance across times. These steps (Steps 0 and 1) are included in the framework in model selection shown in Figure 1.

**LCA and LTA with Covariates**. Researchers may also be interested in examining the effects of covariates on latent statuses and transition between latent statuses. This can be accomplished using multinomial logistic regression. The model can be written as follows

$\delta_{k_{1}}(x)=P(K=k_{1}|X=x)=\frac{\exp(\beta_{0k_{1}}+\beta_{1k_{1}}x)}{1+\sum_{k'_{1}=1}^{K-1} \exp(\beta_{0k'_{1}}+\beta_{1k'_{1}}x)}$ and

$\tau_{k_{t}|k_{t-1}}(x)=P(K=k_{t}|K=k_{t-1},X=x)=\frac{\exp(\beta_{0k_{t}|k_{t-1}}+\beta_{1k_{t}|k_{t-1}}x)}{1+\sum_{k'_{1}=1}^{K-1} \exp(\beta_{0k'_{t}|k'_{t-1}}+\beta_{1k'_{t}|k'_{t-1}}x)}$,

where $k_{t}=1,\cdots,K-1$. Testing the effect of covariates is not limited but often in conflict with estimation problems due to sparseness represented by the ratio of the total sample size (N) and the size of the contingency table (W) (Lanza et al., 2015). As LCA/LTA becomes more complex, sparseness referring to the average expected cell counts becomes smaller, which eventually increases the difficulty in identifying the maximum likelihood solution (Collins & Lanza, 2010).

**A.2 Parameter Estimates**

As in LCA, LTA adopts an iterative approach to parameter estimates consisting of probabilities of latent statuses ($\delta$s), item-response probabilities ($\rho$s), and probabilities of transition ($\tau$s). Either the algorithm of expectation-maximization or Newton-Raphson or a combination of both procedures is used to search for maximum likelihood estimates. Therefore, initial starting values for the estimation are required. Most statistical software, like SAS Proc LTA and Mplus (Muthén & Muthén, 1998-2012), provide either random starting values or user-specific starting values. Regardless of the approach used for the starting values, the algorithm iterates until the convergence criterion or the stopping rules have been reached.

Although full information maximum likelihood (FIML) and multiple imputation (MI) are widely used by researchers in dealing with missing data, both methods have their merits and drawbacks, particularly in LTA models. Specifically, FIML cannot handle missingness when it occurs in grouping variables or covariates in LTA, while with imputed data, the LTA model must be fitted within each imputed data set and then aggregated (Collins and Lanza, 2010). We suggest using FIML as a default option because it is easier to use, unless applied researchers have a strong preference otherwise.

**A.3 Evaluating LTA**

Identifying the number of classes based on the model fit indices is a primary and challenging task in LTA. Both the absolute model fit and relative model fit indices are tools to evaluate the fit of the model. The absolute model fit reflects whether the model adequately represents the data, without reference to competing models (Collins & Lanza, 2010), such as the likelihood-ratio statistic $G^{2}$ compared to the reference $\chi^{2}$ distribution. Conversely, the relative model fit compares two or more models to find an optimal balance of fit to a particular data set and parsimony, such as the likelihood-ratio difference test (LRDT) and information criteria including Akaike Information Criterion (AIC; Akaike, 1974), Bayesian Information Criterion (BIC; Schwarz, 1978), consistent AIC (CAIC; Bozdogan, 1987), and adjusted BIC (ABIC; Sclove, 1987) as in SAS Proc LTA. Due to the asymptotic properties of the likelihood-ratio statistic, the parametric bootstrap likelihood-ratio test (BLRT) is also proposed for LCA (Collins, Fidler, Wugalter, & Long, 1993; Langeheine, Pannekoek, & van de Pol, 1996) and its better performance has been documented (Nylund, Asparouhov, & Muthén, 2007). However, the efficiency of BLRT has not yet been studied for LTA.

In this study, we used AIC and BIC for model comparisons of LTAs. The smaller AIC and BIC, the better the model fit. In the case of disagreement of AIC and BIC, applied researchers may select the optimal number by looking at trends of changes in both. To select the optimal number of latent statuses for student-centered concerns about bullying, for example, we compared LTA models from two-solution to seven-solution by applying both AIC and BIC.

Testing hypotheses about changes of transition probabilities between times were conducted by using likelihood ratio difference test (LRDT). That is, we tested if a single transition matrix would be enough to explain the change in latent statuses across three times. A model with a single transition matrix represents that there is a single pattern of change while a model with two transition matrices represents that the changing pattern from Time 1 to Time 2 is different from the changing pattern from Time 2 to Time 3. The model with a single transition matrix is nested in the model with two transition matrices, therefore, researchers can compare the two nested models using LRDT if this is consistent with their research question.

**Likelihood Ratio Difference Test**. The likelihood ratio statistic, $G^{2}$ (Agresti, 1990), indicating how well a latent transition model fits observed data, is defined as follows

where $f_{w}$ represents the observed frequency of a cell and $\hat{f}_{w}$ represents the expected frequency of a cell according to the model that has been fit. The $G^{2}$ is used to assess the absolute model fit and the larger values of $G^{2}$ provide more evidence that the null hypothesis is not supported. The LRDT is used to compare nested models, for example, Model 2 was nested in Model 1 in our study (See Table A.1). According to the work by Read and Cressie (as cited in Collins & Lanza, 2010), the LRDT statistic, $G_{\Delta}^{2}=G_{2}^{2}-G_{1}^{2}$, with $df_{\Delta}=df_{2}-df_{1}$, is approximated well by the $\chi^{2}$ distribution when $df_{\Delta}$ is relatively small (e.g., < 60, Collins & Lanza, 2010).

To examine measurement invariance, four models were compared to identify the best fitting models in terms of latent status prevalences at Time 1 and transition probabilities (Table A.1). In Model 1, the least restricted model, both latent status prevalences at Time 1 and latent status transitions are free to vary across groups. In Model 2, the latent status prevalences at Time 1 are constrained across groups. In Model 3, the latent status transitions are constrained across groups. In Model 4, both latent status prevalences at Time 1 and latent status transitions are constrained. Comparing these four models allows us to examine the possible variances in terms of latent status prevalences and transition probabilities according to grouping variables. The degrees of freedom of $G^{2}$ increase as model index increases.

To compare Models 1-4, AIC and BIC were also considered in addition to the LRDT because the LRDT works only for nested models, but Models 1-4 are not fully nested. We compared Models 2, 3, and 4 with Model 1 unless either Model 2 or Model 3 fit equally well with the data and fit better than Model 1. When Model 2 or Model 3 fit equally well and fit better than Model 1, we compared Model 4 with Model 2 or 3 via the results of the information criteria, AIC and BIC. Furthermore, the LRDT can also be used to test any constraints. For example, if certain transition parameters should be fixed, the constrained model can be compared with the model with free parameters.

Readers should refer to the supplementary online materials for the specific command files (syntax) using SAS Proc LCA/LTA (PROC LCA & PROC LTA (Version 1.3.2) [Software], 2015) to gain a better understanding of the how to complete the technical steps discussed above.

Table A.1

*Model Comparison of Selecting the Best Fitting Model*

|  |  | Item-Response Probabilities | Latent Status Prevalences at Time 1 | Transition Probabilities |
| --- | --- | --- | --- | --- |
| LTA without grouping variable | Model 1 | Equal across times and latent statuses |  | Free |
|  | Model 2 | Equal across items and latent statuses |  | Equal across times |
| Multiple groups LTA | Model 1 | Equal across times, groups and latent statuses | Free | Free |
|  | Model 2 | Equal across times, groups and latent statuses | Equal across groups | Free |
|  | Model 3 | Equal across times, groups and latent statuses | Free | Equal across groups |
|  | Model 4 | Equal across times, groups and latent statuses | Equal across groups | Equal across groups |

**Appendix B. Syntax for SAS Proc LCA/LTA and Mplus**

**B.1 SAS Proc LCA**

/***********************************************************************/

/***** Step 0: Diagnose and Explore Cross-sectional Data using LCA *****/

/***********************************************************************/

**Proc** **LCA** Data=Jihoonr.bullyingT;

Title1 'Time 1';

NCLASS **2**; /* Number of latent classes: Need to change from 2 to 7 */

ITEMS core2711 core2721 core2731 core2741 core2751 core2761;

CATEGORIES **2** **2** **2** **2** **2** **2**;

/* Stablizing estimates */

GAMMA PRIOR=**1**; /* Only without covariates */

RHO PRIOR=**1**;

seed **741620**;

NSTARTS **20**;

**Run**;

**B. 2 SAS Proc LTA**

/******************************************/

/***** Step 2: Define Latent Statuses *****/

/******************************************/

**Proc** **LTA** Data=Jihoonr.bullyingT;

Title 'Bullying in PRBm with 3 times, 3 statues';

NSTATUS **3**;

NTIMES **3**;

ITEMS core2711 core2721 core2731 core2741 core2751 core2761

core2712 core2722 core2732 core2742 core2752 core2762

core2713 core2723 core2733 core2743 core2753 core2763;

CATEGORIES **2** **2** **2** **2** **2** **2**;

ID id;

measurement times;

seed **741620**;

**Run**;

**B.3 Mplus: LCA**

Title: Step 0: Diagnose and Explore Cross-sectional Data using LCA

Data: File = bullyingT.dat;

Variable: Names = Id core18a1 core18b1 core18c1 core18d1 core18e1 core18f1

core22a1 core22b1 core22c1 core22d1 core22e1 core22f1

core2711 core2721 core2731 core2741 core2751 core2761

core18a2 core18b2 core18c2 core18d2 core18e2 core18f2

core22a2 core22b2 core22c2 core22d2 core22e2 core22f2

core2712 core2722 core2732 core2742 core2752 core2762

core18a3 core18b3 core18c3 core18d3 core18e3 core18f3

core22a3 core22b3 core22c3 core22d3 core22e3 core22f3

core2713 core2723 core2733 core2743 core2753 core2763

Grade Gender Language RaceSch RaceStu;

! Time 1:

Usevar = core2711 core2721 core2731 core2741 core2751 core2761;

Categorical = core2711 core2721 core2731 core2741 core2751 core2761;

Missing = all(-999);

Classes = C1(2); ! Need to change from C1(2) to C1(7)

Analysis: Type = mixture;

Output: Tech11 Tech14;

**B.4 Mplus: LTA**

Title: Step 2: Test Longitudinal Measurement Invariance using LTA

Data: File = bullyingT.dat;

Variable: Names = Id core18a1 core18b1 core18c1 core18d1 core18e1 core18f1

core22a1 core22b1 core22c1 core22d1 core22e1 core22f1

core2711 core2721 core2731 core2741 core2751 core2761

core18a2 core18b2 core18c2 core18d2 core18e2 core18f2

core22a2 core22b2 core22c2 core22d2 core22e2 core22f2

core2712 core2722 core2732 core2742 core2752 core2762

core18a3 core18b3 core18c3 core18d3 core18e3 core18f3

core22a3 core22b3 core22c3 core22d3 core22e3 core22f3

core2713 core2723 core2733 core2743 core2753 core2763

Grade Gender Language RaceSch RaceStu;

Usevar = core2711 core2721 core2731 core2741 core2751 core2761

core2712 core2722 core2732 core2742 core2752 core2762

core2713 core2723 core2733 core2743 core2753 core2763;

Categorical = core2711 core2721 core2731 core2741 core2751 core2761

core2712 core2722 core2732 core2742 core2752 core2762

core2713 core2723 core2733 core2743 core2753 core2763;

Missing = all(-999);

Classes = C1(4) C2(4) C3(4); ! for 4LS model

Analysis: Type = mixture;

Model: %OVERALL%

C2 on C1;

C3 on C2;

Model C1: %C1#1%

[core2711$1] (1);

[core2721$1] (2);

[core2731$1] (3);

[core2741$1] (4);

[core2751$1] (5);

[core2761$1] (6);

%C1#2%

[core2711$1] (7);

[core2721$1] (8);

[core2731$1] (9);

[core2741$1] (10);

[core2751$1] (11);

[core2761$1] (12);

%C1#3%

[core2711$1] (13);

[core2721$1] (14);

[core2731$1] (15);

[core2741$1] (16);

[core2751$1] (17);

[core2761$1] (18);

%C1#4%

[core2711$1] (19);

[core2721$1] (20);

[core2731$1] (21);

[core2741$1] (22);

[core2751$1] (23);

[core2761$1] (24);

Model C2: %C2#1%

[core2712$1] (1);

[core2722$1] (2);

[core2732$1] (3);

[core2742$1] (4);

[core2752$1] (5);

[core2762$1] (6);

%C2#2%

[core2712$1] (7);

[core2722$1] (8);

[core2732$1] (9);

[core2742$1] (10);

[core2752$1] (11);

[core2762$1] (12);

%C2#3%

[core2712$1] (13);

[core2722$1] (14);

[core2732$1] (15);

[core2742$1] (16);

[core2752$1] (17);

[core2762$1] (18);

%C2#4%

[core2712$1] (19);

[core2722$1] (20);

[core2732$1] (21);

[core2742$1] (22);

[core2752$1] (23);

[core2762$1] (24);

Model C3: %C3#1%

[core2713$1] (1);

[core2723$1] (2);

[core2733$1] (3);

[core2743$1] (4);

[core2753$1] (5);

[core2763$1] (6);

%C3#2%

[core2713$1] (7);

[core2723$1] (8);

[core2733$1] (9);

[core2743$1] (10);

[core2753$1] (11);

[core2763$1] (12);

%C3#3%

[core2713$1] (13);

[core2723$1] (14);

[core2733$1] (15);

[core2743$1] (16);

[core2753$1] (17);

[core2763$1] (18);

%C3#4%

[core2713$1] (19);

[core2723$1] (20);

[core2733$1] (21);

[core2743$1] (22);

[core2753$1] (23);

[core2763$1] (24);

Output: Tech1 Tech8 Tech15;
